# Characterization and Analysis of In-Plane Shear Behavior of Glass Warp-Knitted Non-Crimp Fabrics Based on Picture Frame Method

**DOI:** 10.3390/ma11091550

**Published:** 2018-08-28

**Authors:** Ali Habboush, Noor Sanbhal, Huiqi Shao, Jinhua Jiang, Nanliang Chen

**Affiliations:** 1 Engineering Research Center of Technical Textiles, Ministry of Education, Donghua University, Shanghai 201620, China; alitex2008@hotmail.com (A.H.); 1142002@mail.dhu.edu.cn (H.S.); 2 Mechanical Textile Engineering, Aleppo University, Aleppo, Syria; 3 College of Textiles, Donghua University, Shanghai 201620, China; 4 Department of Textile Engineering, Mehran University of Engineering and Technology Jamshoro, Sindh 76062, Pakistan; noor.sanbhal@faculty.muet.edu.pk

**Keywords:** glass fabrics, warp knitting, in-plane shear rigidity, picture frame test, tricot and chain stitches

## Abstract

Glass warp-knitted fabrics have been widely used as complex structural reinforcements in composites, such as wind turbine blades, boats, vehicles, etc. Understanding the mechanical behavior and formability of these textiles is very necessary for the simulation of forming processes before manufacturing. In this paper, the shear deformation mechanics of glass warp-knitted non-crimp fabrics (WKNCF) were experimentally investigated based on a picture frame testing apparatus equipped to a universal testing machine. Three commercially available fabrics of WKNCFs were tested for four cycles by the picture frame method. The aim was to characterize and compare the shear behavior of relatively high areal density fabrics during preform processing for composites. The energy normalization theory was used to obtain the normalized shear force from the testing machine data; then, the shear stress against the shear angle was fitted by cubic polynomial regression equations. The results achieved from the equations demonstrated that the in-plane shear rigidity modulus was associated with the shear angle. The effect of the shearing cycles and stitching pattern on shear resistance was also analyzed.

## 1. Introduction

Recently, increasing importance has been placed on composites and their promising future, according to recent market shares and many predictions [1,2]. The attractive advantage is not just their unique combination of strength and light weight [3], it is also based on the plentiful options for reinforcement and matrix [4]. During the forming process, the textile preforms (whether they are wet or dry) undergo compound in-plane and out-of-plane deformations. Better understanding the mechanical behavior, especially for the relatively new fabrics, can help in many applications, particularly for the forming simulation [5].

There are many deformation modes that can occur in the fabric in the draping process, and the entire deformation can be one or any combination of these modes [6]. Consequently, several tests are required to characterize all of those deformation mechanisms. The most significant mode of deformation is the in-plane shear through the fabric during draping it over double-curvature surfaces [7], and wrinkling is one of the extreme cases during the fabric formation [8]. However, there are different methods to characterize fabric shear resistance, although there is not yet a standard one [9]. The main sources of shear resistance are the in-plane sheer between yarns and the lateral yarn compression [10,11].

### 1.1. Picture Frame (PF) Test

Picture frame (PF) or (trellis shear frame) is a popular apparatus that is essentially adopted to characterize the in-plane shear behavior of textile fabrics [5], especially for dry fabrics and preforms, to produce composites [12,13,14,15,16,17,18,19]. By this simple method of performing testing, pure shear is triggered by the kinematics of the frame [16], which provides reasonably repeatable results if it is carefully implemented, and produces valuable experimental data for analytical and numerical research on two-dimensional (2D) woven fabrics [9], three-dimensional (3D) woven fabrics [12,20], 2D warp-knitted fabrics [15], and 3D spacer knitted fabrics [14]. Moreover, it can be used for unidirectional non-crimp fabrics (UD-NCFs) [16] as well as biaxial non-crimp fabrics (NCFs) [19].

Until now, there has not yet been a standard testing method using the picture frame; there is just a benchmarking effort on the material testing of woven composites fabrics. This effort has proven that the normalization results obtained from this method could be compared to other research results, even if they use different samples and frame sizes [9]. There are three normalization methods reported in the literature: normalizing data by frame length, fabric area, and the energy method. The latter one was proposed by Harrison et al. [21] and developed by Peng et al. [22]. The energy method for normalization has been used because it is the best method for normalizing the shear data of picture frame test to date [9,19].

Lomov et al. demonstrated that the first shear cycle diagrams showed irregular behavior and evidence of tension in the fibers in the frame [23]. Nonetheless, from the second cycle, the sample is mechanically conditioned [22], and the sample behavior becomes much more stable because the fibers were realigned in the frame [19]. Since there is a small difference in the measured force between the second and third cycle, this can significantly improve the repeatability of the test results [9]. 

### 1.2. Warp Knitted Non-Crimp Fabrics (WKNCFs)

Some of the key advantages of knitted fabrics for composite reinforcement over woven ones are: the improvement of fabric handling and matrix injection during composite processing [4], the acceptable processability of high-performance fibers [24], the faster manufacturing of knitted fabrics for reinforcements [25] and controlled anisotropy (capability of in laying yarns in the preferential angle) [4,26]. Non-crimp fabrics can be produced by different techniques [27], but the warp-knitting technique is the most flexible and versatile. WKNCFs have a wide range of areal densities from light to quite heavy fabrics with any staking sequence desired to the application [28].

The essential difference in fabric architecture between NCFs and woven materials makes defect formation mechanisms rather different due to the existence of stitching yarn [10]. So, their behavior should be deeply investigated. In-plane shear is the change of angle between yarns, and is also known as trellising shear [29]. Woven fabrics have been investigated in many studies [9,11,12,17,21,29,30], as have the relatively light carbon NCFs [5,10,16,19], but glass WKNCFs need much more attention for a deeper understanding of their shear behavior, especially with relatively heavy areal densities. Thus, to our knowledge, no work has been done to investigate heavy glass fabrics of about 1200 g/m^2^ using the picture frame.

The objective of this work was to characterize and analyze the in-plane shear behavior of relatively heavy areal density glass WKNCF fabrics based on a picture frame experiment. The in-plane shear rigidity moduli of fabrics were determined for four shearing cycles. For this purpose, a picture frame apparatus was set up, and three types of different glass WKNCFs were tested. The effect of the stitching pattern on the shear deformation mechanism was also observed.

## 2. Materials and Methods

### 2.1. Materials

**Figure 1:**
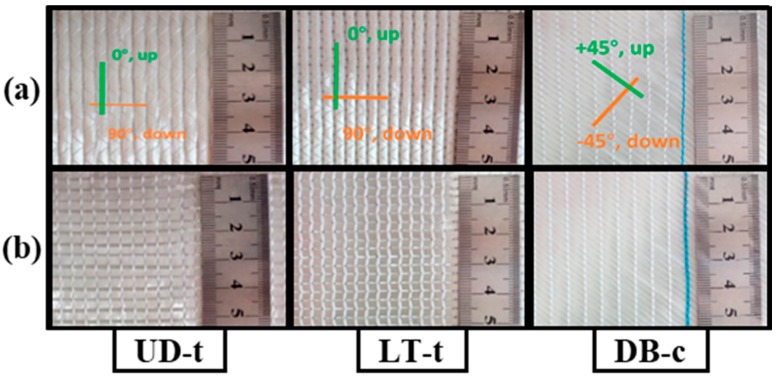
PGTEX^®^ glass warp-knitted non-crimp fabrics (WKNCFs) architecture: (**a**) technical face; (**b**) technical back (colored figure in the web version of this article).

Glass warp-knitted non-crimp fabrics (glass-WKNCFs) from PGTEX^®^ company (Changzhou, China; product codes TM-L1250-7, E-LT1100-7, and E-DB1200-6) were used for the experiments in this study. Fabrics with heavy areal densities (about 1200 g/m^2^) and typical stitching patterns (tricot for UD (unidirectional) and LT (longitudinal-transverse) and chain stitching for DB (double-bias)) were characterized here. The photographs of NCF fabrics on both faces are presented in Figure 1.

The fabrics were made from more than one set of yarns. Each yarn set (longitudinal, transverse, and bias directions) was approximately straight in its layer in the fabric plane. The stitching yarn with smaller linear density connected the layers through the fabric thickness and held adjacent layers together. The other fabric details were listed in Table 1, and the roving properties and fabric manufacturing details are shown in Appendix A. The three fabrics had two layers with different parameters, but for UD, the one on the technical back was only to support the structural cohesion and facilitate the manufacturing process [16], and it was just about 6% of the total areal density. The fabrics had approximate areal densities (~10% difference for LT-t).

**Figure 2:**
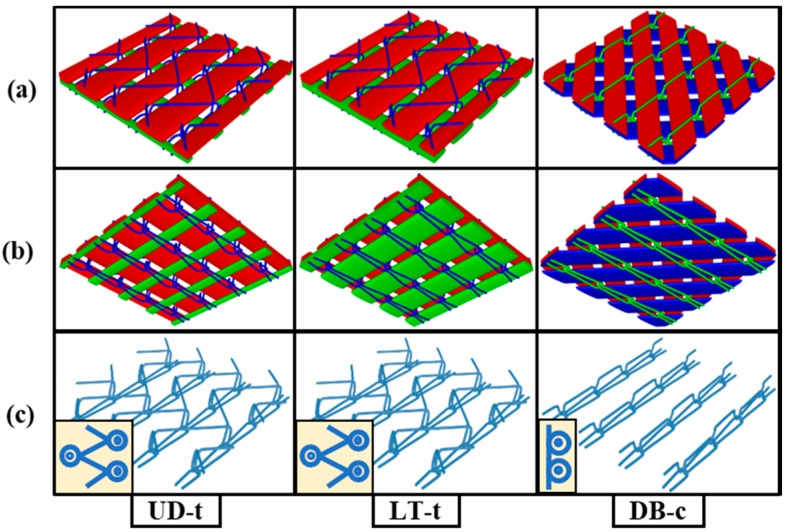
Three-dimensional (3D) geometric model of fabric structures by TexGen software: (**a**) technical face; (**b**) technical back; and (**c**) the stitching pattern and its notation at the corner.

The naming system abbreviations used by the manufacturing company for these fabrics are as following: UD denotes unidirectional, L denotes the longitudinal orientation of the fabric (0°), which is the machine production direction, T stands for the transverse orientation (90°) or cross-machine direction, and DB denotes the double-bias where the yarns are at a specific angle from the machine production direction, which is mainly (+45°/−45°). According to this naming system, the codes UD, LT, and DB become clear. The small letter “t” stands for tricot stitching, and “c” stands for chain stitching; these were added to the original name of the fabric for easily distinguishing the stitching type associated with each fabric. Moreover, all of the other fabric details are listed in Table 1. The fabrics’ 3D geometry was modeled by TexGen software (textile geometric modeler, TexGen 3.5.0) [31]. Both sides and the stitching patterns are shown in Figure 2.

### 2.2. Methods

**Figure 3:**
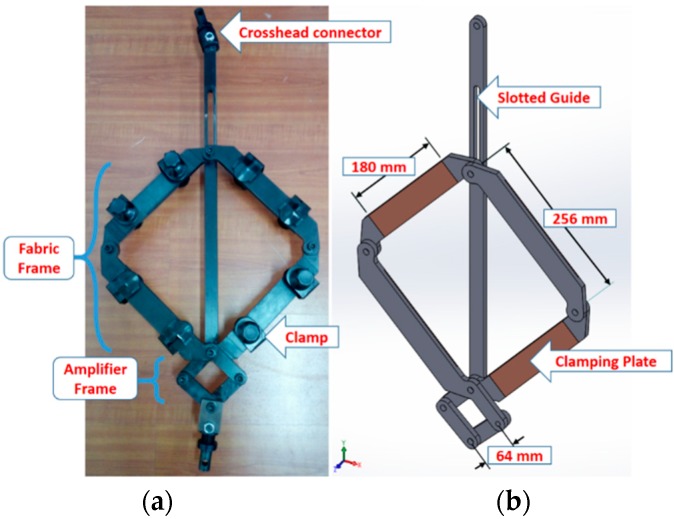
The picture frame: (**a**) the manufactured frame with the clamps and connectors; (**b**) a SOLIDWORKS 3D design.

Studying the deformation mechanisms of fabrics, especially shear behavior by picture frame, needs a lot of attention in order to be done carefully. For all of the material characterization tests described in the following sections, a universal testing machine (WDW-20, Shanghai Hualong Test Instrument Co., Ltd., Shanghai, China) provided by the Engineering Research Center of Technical Textiles was used. The frame was mounted in a load cell (WDW-20, Shanghai Hualong Test Instrument Co., Ltd., Shanghai, China) for measuring the amplifier frame displacement force, as shown in Figure 3. All of the tests were performed at room temperature and a constant crosshead displacement rate of 10 mm/min. Hence, the strain rate of fabric shearing increased as the amplification factor of the device increased. The specimens of each type of fabric were tested in the frame for four cycles. At least three repeats should be conducted under identical conditions, according to Harrison [32], and the average values of three repeats for each fabric were presented in the results.

The manufactured picture frame for this study was used to test in-plane shear behavior on cruciform fabric samples, where the length of the fabric was less than that of the frame (*L*_fabric_ < *L*_frame_) in order to avoid corner buckling. The design of the frame apparatus resembled that used by UML (University of Massachusetts Lowell, Lowell, MA, USA,) [14], but with a slight difference in dimensions. The design details and dimensions are illustrated in Figure 3.

The slotted guide (sliding link) had two functions: the first one was connecting one corner of the small amplifier frame to the crosshead of the tensile testing machine (WDW-20, Shanghai Hualong Test Instrument Co., Ltd., Shanghai, China), and the second one was to limit the fabric frame motion between the starting position and final deformed position i.e., between 0° and 60° (1.05 rad) of shear angle. This frame was connected to the crossheads of the tensile machine by two connectors. In addition, an extra two connectors were manufactured in order to use the same frame to other tensile testing machines. The amplifier itself would be helpful if the crosshead displacement and/or the crosshead velocity of the testing machine is limited.

**Figure 4:**
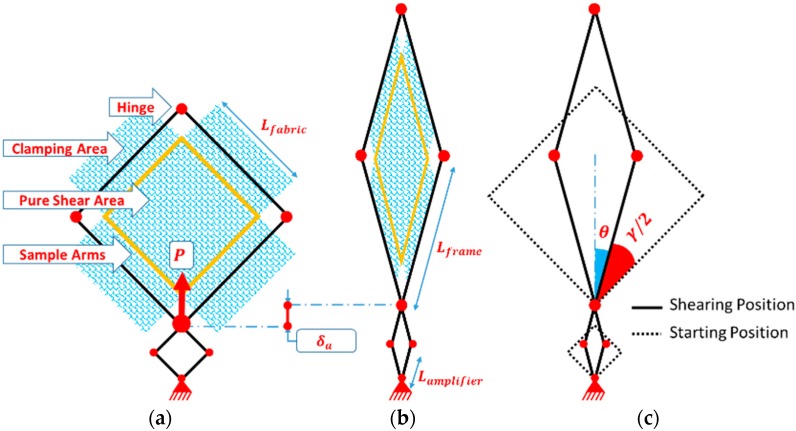
Schematic diagram of the frame design: starting position (**a**); shearing position (**b**); and shear angle (**c**).

From the illustrations in Figure 4, when the amplifier frame is deformed from the starting position by δ_a_ displacement due to applying the tensile force P, the fabric frame is deformed by αδ_a_, where α is the device amplification factor; it can be calculated by the following equation:

(1) $$\alpha =\frac{L_{\mathrm{frame}}}{L_{a}}=\frac{256\left[\mathrm{mm}\right]}{64\left[\mathrm{mm}\right]}=4$$

The power applied to the frame P is dissipated in the shearing zone. By simple kinematic analysis of the frame, shear angle γ and be given as a function of the amplifier frame displacement δ_a_ by Equation (3):

(2) $$\theta =\cos^{-1}\left(\frac{L_{a}\sqrt{2}+\delta_{a}}{2L_{a}}\right)=\cos^{-1}\left(\frac{\sqrt{2}}{2}+\frac{\delta_{a}}{2L_{a}}\right)$$

(3) $$\gamma =\frac{\pi }{2}-2\theta =\frac{\pi }{2}-2\cos^{-1}\left(\frac{\sqrt{2}}{2}+\frac{\delta_{a}}{2L_{a}}\right)$$

where a subscript notation denotes amplifier.

**Figure 5:**
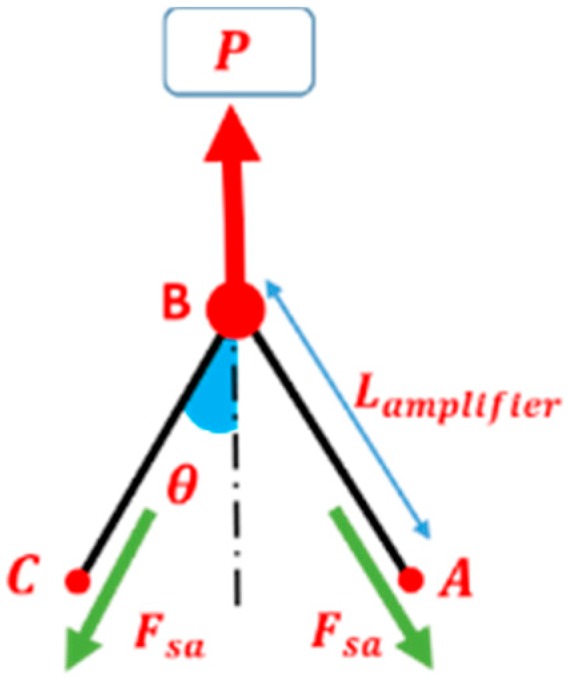
Free body diagram of the amplifier shear frame.

**Figure 6:**
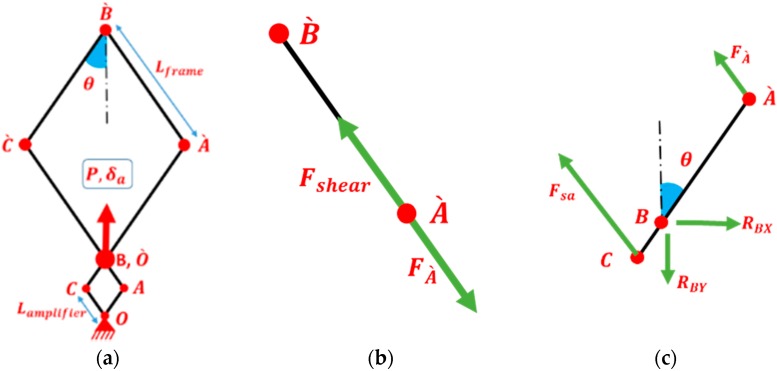
Schematic diagram of the frame: (**a**) shearing position at θ; (**b**) free body diagram of $\overset{ˋ}{A}\overset{ˋ}{B}$; and (**c**) free body diagram of the linkage $CB\overset{ˋ}{A}$.

From the free body diagram of the amplifier frame as shown in Figure 5 and Figure 6a, the shear force of the amplifier can be calculated:

(4) $$F_{\mathrm{sa}}=\frac{P}{2\cos\theta }$$

where P is the force measured by the load cell in the crosshead.

The free body diagrams of the link fabric frame $\overset{ˋ}{A}\overset{ˋ}{B}$ and $CB\overset{ˋ}{A}$ are shown in Figure 6b. The hinge $\overset{ˋ}{A}$ is free to move. From geometrical symmetry, it can be determined that the force applied on joint $\overset{ˋ}{B}$ from links $\overset{ˋ}{C}\overset{ˋ}{B}$ and $\overset{ˋ}{A}\overset{ˋ}{B}$ is zero. Therefore, by a static analysis using the free body diagram of link $\overset{ˋ}{A}\overset{ˋ}{B}$, it can be concluded that:

(5) $$F_{\mathrm{shear}}=F_{\overset{ˋ}{A}}$$

where $F_{\overset{ˋ}{A}}$ is the force on joint $\overset{ˋ}{A}$ between link $\overset{ˋ}{A}\overset{ˋ}{B}$ and link $CB\overset{ˋ}{A}$, and $F_{\mathrm{shear}}$ is the shear force applied to the fabric sample by the link $\overset{ˋ}{A}\overset{ˋ}{B}$. Then, from the free body diagram of link $CB\overset{ˋ}{A}$, $\sum M_{B}=0$:

(6) $$F_{\overset{ˋ}{A}}L_{\mathrm{frame}}\sin\left(2\theta \right)-F_{\mathrm{sa}}L_{a}\sin\left(2\theta \right)=0$$

where $M_{B}$ is the moment at point B, $L_{\mathrm{frame}}$ is the length of link $\overset{ˋ}{A}\overset{ˋ}{B}$, $F_{\mathrm{sa}}$ is the shear force applied on the amplifier frame from the tensile machine, and θ is the angle between link $\overset{ˋ}{A}\overset{ˋ}{B}$ to the vertical direction.

Solving Equation (6) for $F_{\overset{ˋ}{A}}$, and according to Equation (1) by including the amplification factor:

(7) $$F_{\overset{ˋ}{A}}=\frac{F_{\mathrm{sa}}L_{a}}{L_{\mathrm{frame}}}=\frac{F_{\mathrm{sa}}}{\alpha }$$

By substituting in Equation (4), the net shear force applied on the fabric can be obtained by:

(8) $$F_{\mathrm{shear}}=\frac{P_{\mathrm{load}}-P_{\mathrm{empty}}}{2\alpha \cos\theta }$$

Since the fabric sample is crucified, the sheared region by frame is square (180 × 180 mm^2^) with four-sided arms connecting it to the clamping areas in the frame. The 2D geometrical details of the frame are illustrated in Figure 4. The kinematics of the test leads to a perfect pure shear zone in the fabric sample, assuming that the yarns are non-extensible, and there is no slippage within the sample [6].

The two principal orientations of the fibers in the sample are connected to the clamping areas of the frame, so that the frame can be used to characterize only unidirectional and biaxial fabrics, not triaxial or quadriaxial fabrics.

### 2.3. Test Setups

The fabric samples were fixed in the frame under zero tension, because zero pre-tension eliminated the tension–shear coupling and gave the most accurate response [33].

**Figure 7:**
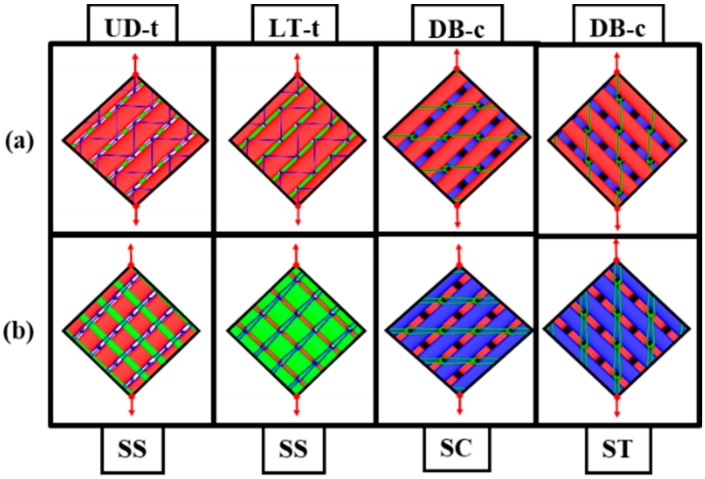
Schematic diagrams of fabric samples’ arrangement inside the frame: (**a**) technical face; (**b**) technical back. SS = stitch-shear, SC = stitch-compression, ST = stitch-tension.

The fabric samples’ orientation in relation to the fabric frame sides was illustrated in Figure 7. Since the fabric specimens with the tricot stitch pattern (LT) were symmetrical in shearing behavior, there was no difference in shearing behavior related to the rovings orientation inside the frame (SS = stitch-shear [19]). However, this case was not the same for the DB fabric due to the chain stitch pattern; the shearing was asymmetrical according to the chain stitch orientation in relation to the shearing direction. On one shearing direction, the stitches were under compression (SC = stitch-compression [19]) during the shear cycle, whereas in the other shearing direction, the stitches suffered from tension (ST = stitch-tension [19]). So, that DB chain stitch was tested in two directions separately. One set of samples was tested in the stitch-compression direction (SC), and the other set was tested in the stitch-tension direction, as shown in Figure 7.

Special attention should be taken to make sure that the fiber yarns were orientated properly to the edges. Any small misalignment will produce tensile or compressive forces in the fiber directions, and as a consequence, a large scatter in the measured force readings will appear [21]. However, the first shear cycle for fabrics showed extremely irregular behavior and evidence of tension in the fibers in the frame [19]; this type of behavior was labeled in [19,34] as ‘bad tests’, and normally, such tests were discarded from the data processing. In addition, when repeating the test for the same fabric, we came up with some extreme irregular cases from the general trend, which were not included in the analysis in this paper.

### 2.4. Testing Protocol (Data Processing, Normalization, and Fitting)

**Figure 8:**
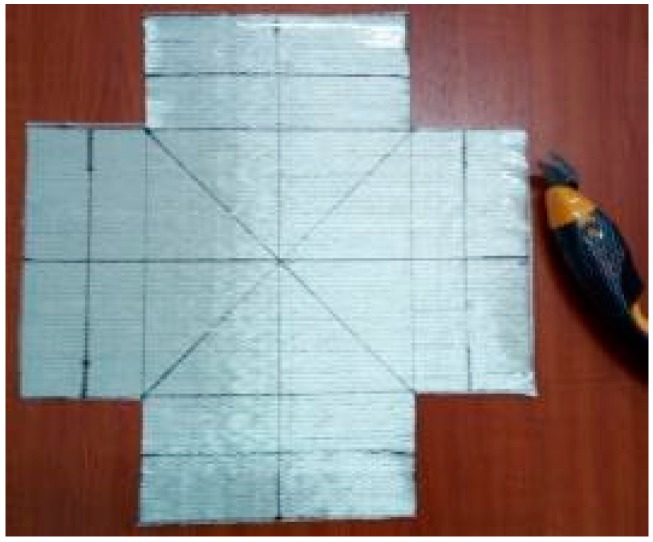
A sample of UD-t after cutting by “e-scissor” with the marking lines.

In the beginning, a piece of fabric was cut from the roll carefully, and the marking lines were drawn by a metallic ink marker pen (suitable for glass fibers). The cutting of samples was done by “electric scissor” (shown in Figure 8) to ease the cutting process, prevent the normal problems of using ordinary scissors, such as fiber slippage and local pull-out, and consequently reduce preparation misalignments and increase test reproducibility.

Firstly, the frame was tested when it was empty, and the displacement-force data were recorded. Then, the fabric samples were fixed in the frame with zero tension, and the displacement-force data for the frame and fabric together were recorded. Then, the net shear force can be calculated by Equation (8) above.

However, this force cannot be directly compared to the results from the other picture frame arrangements or to the results from the other shear behavior characterization methods (similar to UBE (uniaxial bias extension). However, the benchmark effort [9] referred to the capability of comparing different picture frame results by using a normalization method. According to the researchers [21,22], normalization of the shear force based on the energy approach was the best method for the picture frame test. It had been used when the length of the fabric sample was not necessarily equal to the length of the frame, similar to the situation that we had in this study. So, the shear force data could be normalized using the following equation, which was also used by [10]:

(9) $$F_{\mathrm{normalized}}=F_{\mathrm{shear}}\times \frac{L_{\mathrm{frame}}}{L_{\mathrm{fabric}}^{2}}$$

From this equation, the normalized shear force was a function of the frame shear angle (θ), and in order to calculate the shear stress, we simply divide the normalized shear force by the thickness of the fabric:

(10) $$\tau =\frac{F_{\mathrm{normalized}}}{t}$$

where *t* was the thickness of the fabric, assuming the fabric thickness was constant during the test [6]. The measured thicknesses according to the ASTM D1777-2015 “Standard test method for thickness of textile materials” [35] are given in Table 2.

The cubic polynomial function was chosen; it had potential benefits within a finite element model, and its derivative can be easily calculated and implemented into a mode [11]. By fitting the shear stress curves using polynomial cubic fitting from the origin point (start from (0,0)), the expression of shear stress as a function of shear angle can be obtained [using fitting in this stage is more accurate]:

(11) $$\tau \left(\gamma \right)=A+B\gamma +C\gamma^{2}+D\gamma^{3}$$

where *A* = 0; then, the shear modulus *G*_12_ can be calculated from the derivative of the fitting equation determined from the data points on the curve of the shear stress versus the shear angle; the shear angle unit is in radians.

(12) $$G_{12}\left(\gamma \right)=B+2C\gamma +3D\gamma^{2}$$

In our measurement for WKNCFs, the tricot stitch pattern for biaxial fabric could be mechanically conditioned’, and it could be experimentally proved that the second and third cycle were much more stable in characterizing the in-plane shear behavior. On the other hand, the chain stitch pattern for biaxial fabric could be conditioned just in the SC direction, but not in the ST direction, because some stitches will be broken in the first cycle. Still, the next shear cycles were implemented on the fabric to investigate the change in shear behavior.

## 3. Experimental Results

In this study, four shearing cycles were executed for each fabric sample. For each cycle, diagrams during the loading phase were registered, while the diagram during the unloading phase (returning the frame to the starting position) were not registered.

**Figure 9:**
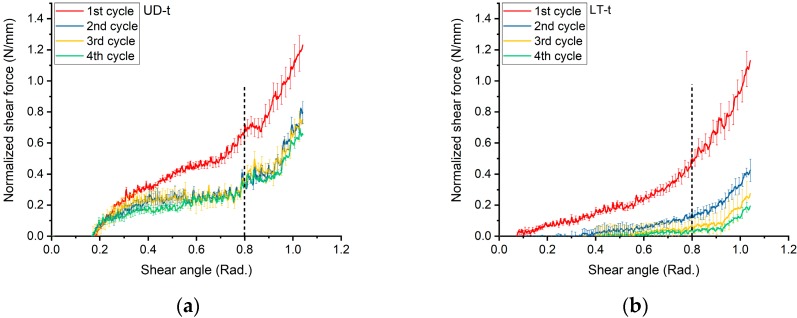

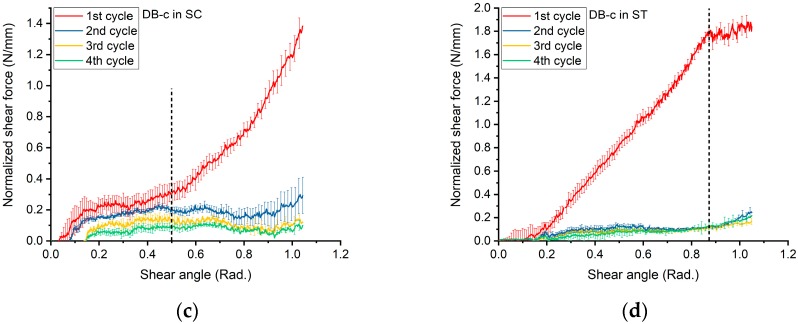
Normalized shear force: (**a**) UD-t in SS = stitch-shear; (**b**) LT-t in SS = stitch-shear; (**c**) DB-c in SC = stitch-compression; and (**d**) DB-c in ST = stitch-tension. Error bars indicate standard deviation in three tests.

”Bad tests” data were discarded from the processing, which indicates the test with extremely irregular behavior and the evidence of tension of the fibers in the frame. The normalized shear force for fabrics is given in Figure 9.

Considering the thickness of fabric samples, the shear stress curves against the shear angle would be obtained. By fitting these curves using cubic polynomial regression, the values of Equations (11) and (12) would be obtained. The details were shown in Appendix B.

**Figure 10:**
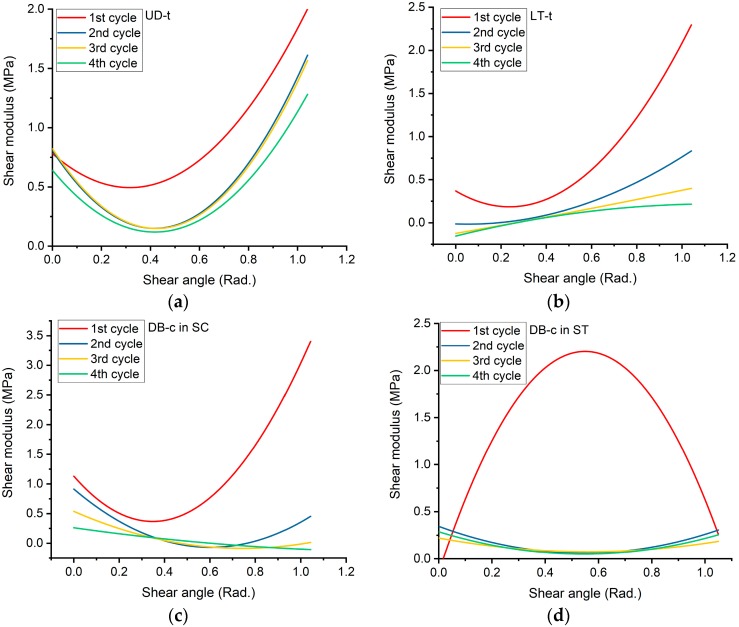
In-plane shear rigidity modulus: (**a**) UD-t in SS = stitch-shear; (**b**) LT-t in SS = stitch-shear; (**c**) DB-c in SC = stitch-compression; (**d**) DB-c in ST = stitch-tension.

Then, by the derivation of these curves, the shear modulus *G*_12_ has been determined. The curves were illustrated in Figure 10, and the values of the equation coefficients are provided in Appendix B.

From the curves in Figure 10, for the tricot pattern, the fabric shear rigidity generally increased with the shear angle and had a similar trend in all of the cycles. Besides, for the chain stitching pattern under compression, the shear modulus had the same trend as the tricot pattern in the first cycle, and decreased in the following cycles. However, for chain stitching in tension, the shear modulus increased and then decreased rapidly after some stitches were broken.

## 4. Discussion

### 4.1. Shear Deformation Mechanics and Effect of the Shearing Cycles

#### 4.1.1. Unidirectional with Tricot Stitch Fabric in Stitch Shear (UD-t in SS)

Figure 9a presented the curves of the normalized shear force (expressed in units of N/mm) against the shear angle (in radian) for UD-t fabric. The chart of the first shear cycle showed the same trend as the next shearing cycles, which means that there was no evidence of fiber tension inside the frame; thereby, zero pre-tension was achieved. The shear behavior in the second, third, and fourth cycles was almost the same due to the small differences in the shear force among the cycles. Therefore, the tricot pattern induced the same effects on the shear mechanisms by different cycles.

Essentially, the graphs started from the origin, but generally, the WKNCF fabrics were easy to deform. At the first stage of shearing (~0.17 rad), the curves were near to the zero, because the applied force was just making rovings to be closer to each other. Consequently, the spaces between rovings were diminishing without any lateral compaction. Then, in the next stage, which starts from (~0.17 rad) of shearing angle, the lateral compaction between neighboring rovings was arising gradually up to ~0.26 rad. After that, a rapid rise in the load resistance of the fabric sample was observed because rovings began to squeeze within the layer. However, the effect of the transverse roving layer was neglected.

**Figure 11:**
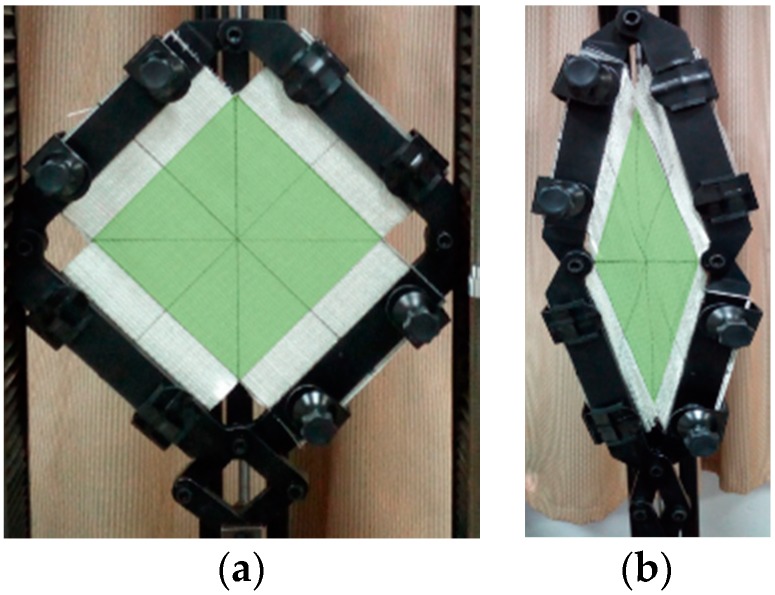
UD-t sample in the picture frame apparatus; the region subjected to pure shear is in green. (**a**) Starting position; (**b**) at 1.05 rad shearing angle (colored figure in the web version of this article).

**Figure 12:**
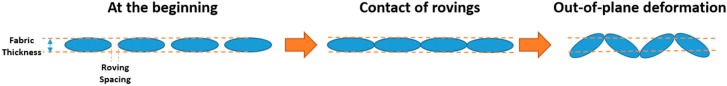
Schematic diagram of local roving buckling due to lateral compaction.

From visual observations, between the starting and final position, as shown in Figure 11, there were no wrinkles up to 1.05 rad of the shear angle, but there was local out-of-plane buckling due to the lateral compaction in the sheared region, as illustrated in Figure 12.

#### 4.1.2. Biaxial 0°/90° Tricot Stitch Fabric in Stitch Shearing (LT-t in SS)

At the initial stage of shearing, the normalized shear force was very low due to the free rotation of rovings inside the tricot pattern, as a curve was shown in Figure 9b. Then, the shear force increased gradually as the spacing between the rovings was vanishing, and the compaction between them start to occur, causing the force to increase rapidly. However, in the next shearing cycles, the fabric became easier to be sheared because of the mechanical conditioning or “softening” of the tricot structure. Having a similar trend of shear force by the next cycles was evidence that the zero pre-tension was achieved. Moreover, the values of the shearing force in the following cycles were lower because of the strained tricot pattern in the first cycle.

**Figure 13:**
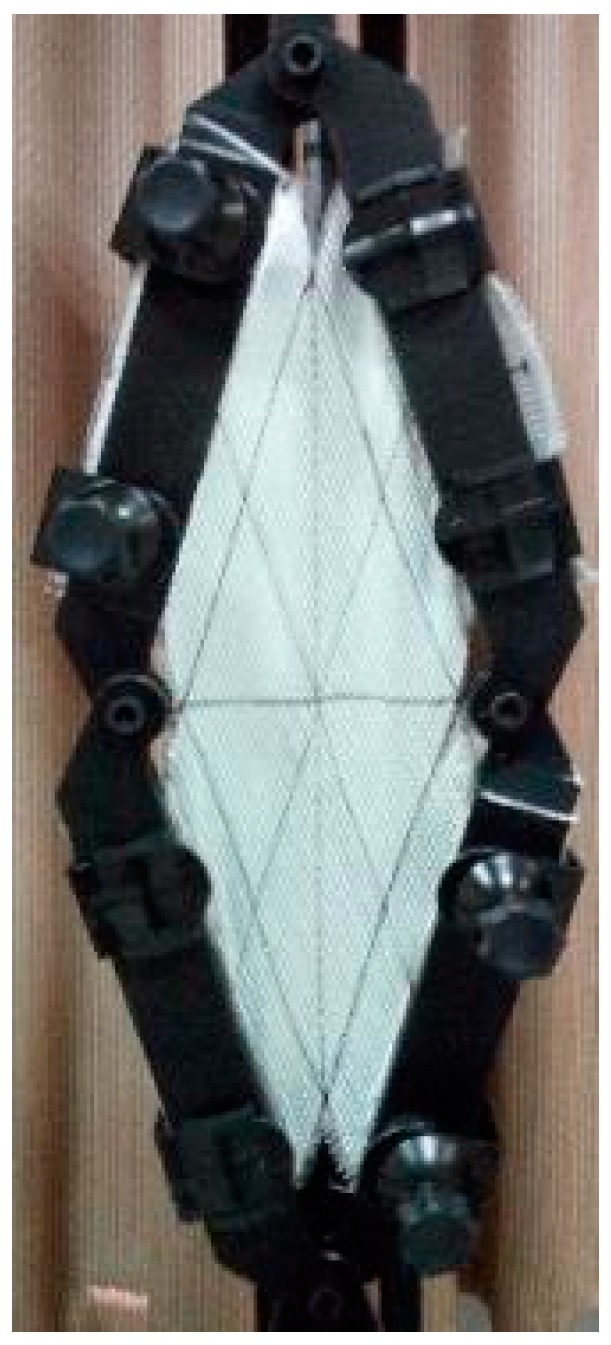
LT-t in the deformed position sample (1.05 rad shearing angle).

In the comparison of shear behavior between LT-t and UD-t fabric, there were no out-of-plane wrinkling, and even no local buckling occurred in LT-t fabric until the end of the shearing test (~1.05 rad), as in the photograph in Figure 13. The distribution of fabric weight between the layers was the reason for this difference in wrinkle formation. In the LT-t fabric, the areal density was distributed between the longitudinal and transverse layer nearly in an equal manner, but the weight in the UD-t fabric was mostly in just one layer. Therefore, the LT-t structure had more spaces between the rovings in each layer. Consequently, it was easier to be deformed by shearing, which was apparent in the curves of Figure 9a,b.

#### 4.1.3. Biaxial +45°/−45° Chain Stitch Fabric in Stitch Compression (DB-c in SC)

For biaxial chain stitch fabric +45°/−45°, the chain-stitching pattern made the fabric behavior asymmetrical in shearing. In this case, the chain stitch would suffer from compression in one direction and from tension in the other direction. Thus, different modes of deformation were observed in the stitch compression direction for the DB-c fabric. The +45° and −45° rovings rotated toward the loading direction and therefore, they were forced to slide through the stitch loops. This sliding led to frictional resistance between the stitches and rovings. The sliding of rovings as they rotated was the reason for the low load resistance.

**Figure 14:**
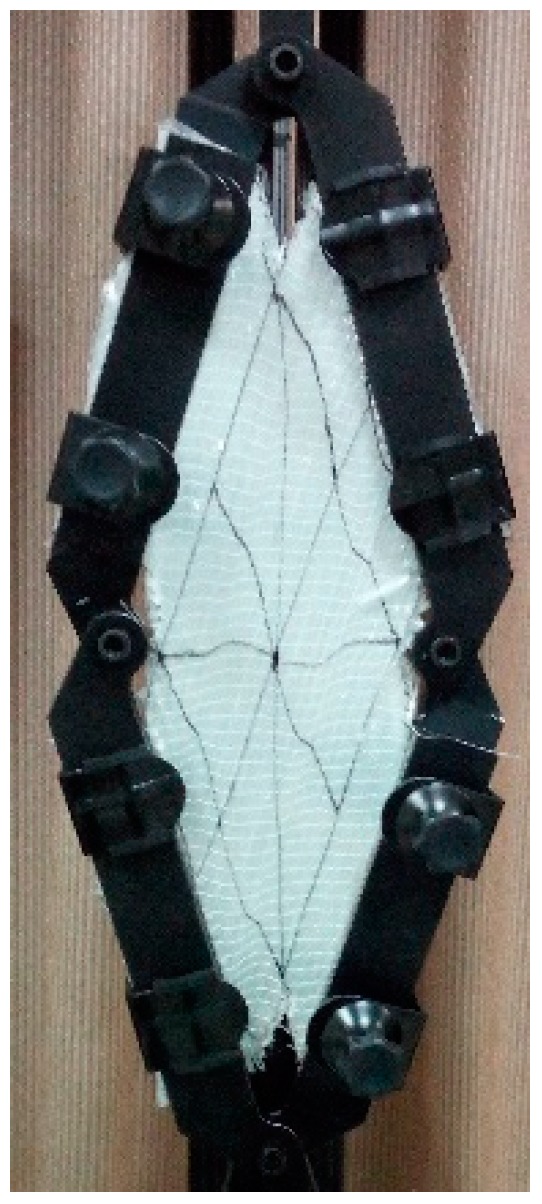
DB-c sample at a 1.05 rad shearing angle.

In the curve of the first cycle in Figure 9c, the normalized shear force started to rise gradually up to ~0.5 rad. The rovings became close to each other due to rotating and sliding between rovings inside the stitches. After 0.5 rad, the lateral compaction led to a rapid increase in the shear force and the rovings would be squeezed together within the fabric. This action would not break the stitches in the chains, but it was responsible for the rapid increase in the load resistance. However, in the next cycles, there was just a slight increase in the shear force within the cycle, which means that the stitches were strained by the compressed rovings during the first cycle, and became “mechanically conditioned” or “softer”, which have made the resistance lower in the next cycles. In addition, from visual observations, there were no wrinkles up to 1.05 rad of the shear angle, as shown in Figure 14. Only small local buckling in some rovings occurred at a large deformation angle due to the restrictions of chain stitches during the compaction.

#### 4.1.4. Biaxial +45°/−45° Chain Stitch Fabric in Stitch Tension (DB-c in ST)

**Figure 15:**
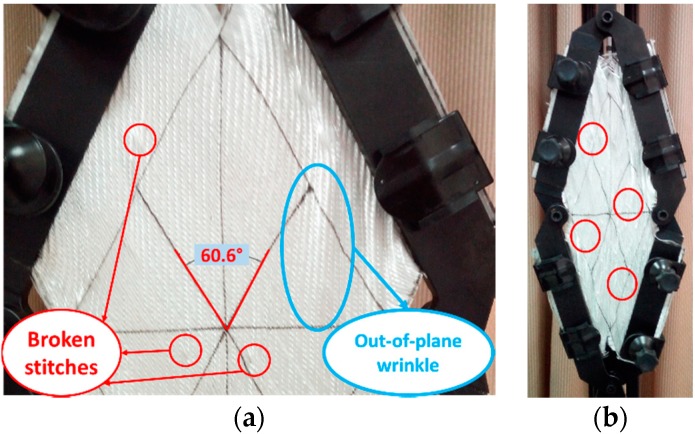
DB-c in ST: (**a**) at ~0.5 rad; (**b**) at 1.05 rad.

A different mechanism of deformation was detected in stitch tension direction for the DB-c fabric; where the direction of loading made the stitches under tension, this arrangement applied very high fixation to the rovings. Therefore, additional rotations were limited, and global out-of-plane wrinkling could be observed at an earlier stage in comparison to stitch compression (SC), as shown in Figure 15. Continuing the test would result in more noticeable wrinkles, especially near the center of the sheared zone. Stitch tension was the reason for the steep increase of the shearing force, up to an ~0.87 rad shear angle. This fast increase of the shear force could be clarified by tensile modulus stitching chains being several times higher than the friction and compaction modulus of the rovings [19]. Then, the shear force would not be increased, because so many stitches would be broken, and there was nothing holding the rovings together, as the curves were shown in Figure 9d.

The first break of stitches under tension appeared at an ~0.5 rad shear angle, as illustrated in Figure 15a. After that, many local wrinkles appeared between the unbroken loops, as shown in Figure 15b.

By comparing the shear force between the two directions for the same fabric, it could be concluded that the shear behavior was anisotropic. This matter should be considered in establishing a good material model for such type of fabrics in order to accurately predict their total shear behavior.

For the samples of UD-t, LT-t, and DB-c in SC, the in-plane shearing behavior could be analyzed from the second cycle, since the second cycle reflected the pure in-plane shear behavior, as many studies considered [9,19]. However, for DB-c in ST, just the first cycle should be analyzed to investigate the shear behavior in the stitch tension direction, due to the breakage of some stitches during the first cycle of shearing.

### 4.2. In-Plane Shear Rigidity Modulus

From the mentioned procedure in Section 2.2 to determine the in-plane shear rigidity modulus expression as a function of the shear angle in radians, the following equations can be written for the first cycle of UD-t, LT-t, DB-c-SC, and DB-c-ST, respectively:

(13) $$G_{12}{\left(\gamma \right)}_{\mathrm{UD}}=0.23078+0.69608\gamma -0.13284\gamma^{2}$$

(14) $$G_{12}{\left(\gamma \right)}_{\mathrm{LT}}=0.36923-1.54978\gamma +3.26637\gamma^{2}$$

(15) $$G_{12}{\left(\gamma \right)}_{\mathrm{BD}\;\mathrm{in}\;\mathrm{SC}}=1.13072-4.38108\gamma +6.28908\gamma^{2}$$

(16) $$G_{12}{\left(\gamma \right)}_{\mathrm{DB}\;\mathrm{in}\;\mathrm{ST}}=-0.1446+8.53802\gamma -7.76553\gamma^{2}$$

These equations have been plotted, and the values of coefficients for all of the cycles are given in Appendix B.

## 5. Conclusions

The in-plane shear rigidity modulus for glass WKNCFs fabrics based on picture frame test data were evaluated. The procedure was just depending on the picture frame shear experiment. The force-displacement results during the tests were used to obtain the normalized shear force, shear stress, and in-plane shear rigidity modulus. This modulus was a very important feature in characterizing the mechanical behavior of the reinforcement fabrics for forming the simulation of composites. The simple procedure of defining the shear modulus equation was presented based on the pure shear mechanism of the picture frame apparatus.

Three types of warp-knitted non-crimp fabrics (WKNCFs) were investigated. The fabrics had two different stitching patterns, namely, tricot and chain. The cubic polynomial approximation was used to determine the shear stress equation against the shear angle; then, shear rigidity had been calculated from the derivative of the shear stress fitting equations. It was also found that the shear rigidity for fabrics with a tricot pattern increased with the shear angle, and showed the same trend in cycles with a lower shearing force. For the “chain stitching pattern under compression”, the shear rigidity was decreased in the initial stage of shearing, and afterwards, it increased rapidly in the first cycle, while it generally decreased in the next cycles. On the other hand, for the “chain stitching pattern under tension”, the shear rigidity increased up to the stage of some stitches being broken, at which point it decreased rapidly.

## Figures and Tables

**Table 1:** Summary of PGTEX^®^ glass WKNCF specifications.

Specification	Unidirectional 0°	Biaxial 0°/90°	Biaxial +45°/−45°
Material designation	L1250-7	LT1100-7	DB1200-6
Fabric areal density (g/m^2^)	1250 (1236) * (0° = 1156/90° = 80)	1100 (1116) * (0° = 606/90° = 491)	1200 (1235) * (45° = 601/−45° = 601)
Stitch yarn	Polyester	Polyester	Polyester
Linear density of stitching yarn (*D*)	100	100	100
Stitch pattern (face–back)	Tricot-chain	Tricot-chain	Chain-Chain
Stitch areal density (g/m^2^)	12	11	7
Material code in this paper	UD-t ^1^	LT-t ^2,3^	DB-c ^4^

^1^: UD = Unidirectional; ^2^: L = Longitudinal; ^3^: T = Transverse; ^4^: DB = Double-Bias; t = tricot, c = chain; and *: measured areal density.

**Table 2:** Fabric thicknesses.

Fabric Code	*t* (mm)
UD-t	1.25
LT-t	1.36
DB-c	1.2

**Figure A1:**
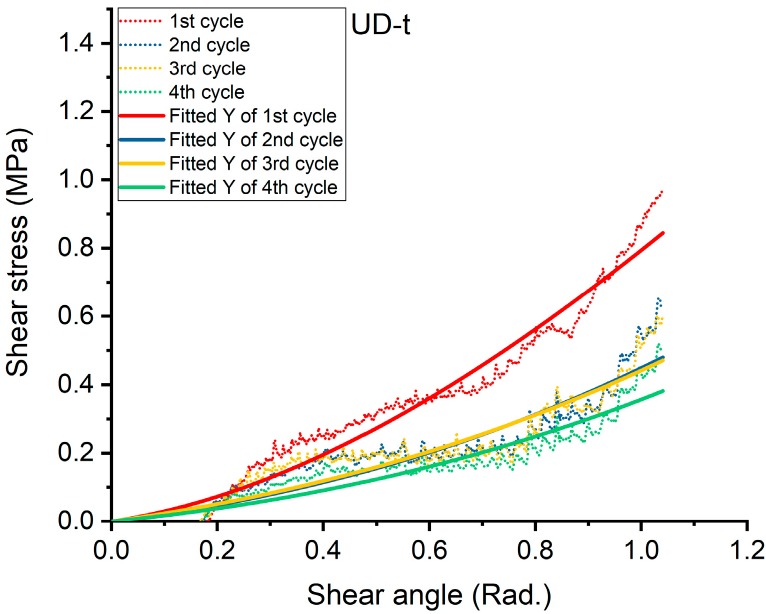
Shear stress curves for four cycle of UD fabric.

## Appendix A

**Table A1:** Fabric manufacturing details and roving properties.

Specification	Uni-Directional 0°	Bi-Axial 0°/90°	Bi-Axial +45°/−45°
Material designation	L1250-7	LT1100-7	DB1200-6
Machine gauge	E5	E5	E5
Run-in length per rack (m)	6.9	5.5	4.6
Course density per cm	2.5	3.5	4.6
Wale density per cm	2.8	2.8	1.9
Stitch length (mm)	14.4	11.5	9.6
Material code in this paper	UD	LT	DB

Roving tensile strength = 0.46 N/tex according to GB/T 7690.3 (ISO 3341) testing standard [36], and filament diameter = 17 μm according to GB/T 7690.5 (ISO 1888) [37].

## Appendix B

### Fitting Data of Stress Curves

For each fabric and each cycle, the average shear force was calculated, and then used to find the shear stress. The shear stress was fitted using OriginPro^®^ software (2017) into a cubic polynomial function, and the coefficients with their corresponding standard error for all of the fabrics and cycles are given in Table A2 below.

**Table A2:** Cubic polynomial regression coefficients of all of the fabrics and cycles in this study.

Cubic Polynomial Regression Shear Stress (MPa) Versus Shear Angle (Radian)				
Equation	*y = A + Bx + Cx* ^2^ *+ Dx* ^3^			
**Model**	**UD-t in SS**			
Plot	1st cycle	2nd cycle	3rd cycle	4th cycle
*A*	0	0	0	0
*B*	0.23078	0.19699	0.21942	0.15659
*C*	0.69608	0.20446	0.17528	0.16253
*D*	−0.13284	0.04723	0.04658	0.03754
Reduced Chi-Sqr.	5.16 × 10^−3^	5.95 × 10^−3^	6.30 × 10^−3^	3.76 × 10^−3^
*R*-Square (COD *)	0.92309	0.78099	0.76775	0.78099
Adj. *R*-Square	0.92303	0.78082	0.76757	0.78082
**Model**	**LT-t in SS**			
Plot	1st cycle	2nd cycle	3rd cycle	4th cycle
*A*	0	0	0	0
*B*	0.36923	−0.01327	−0.12329	−0.15442
*C*	−0.77489	−0.04628	0.22919	0.32619
*D*	1.08879	0.29004	0.01397	−0.09504
Reduced Chi-Sqr.	5.49 × 10^−4^	4.66 × 10^−4^	5.04 × 10^−4^	4.69 × 10^−4^
*R*-Square (COD *)	0.98362278	0.905371738	0.752072267	0.618709869
Adj. *R*-Square	0.983609879	0.905297198	0.751876971	0.618409523
**Model**	**DB-c in SC**			
Plot	1st cycle	2nd cycle	3rd cycle	4th cycle
*A*	0	0	0	0
*B*	1.13072	0.91366	0.53815	0.26212
*C*	−2.19054	−1.62744	−0.83705	−0.27092
*D*	2.09636	0.89917	0.37458	0.06045
Reduced Chi-Sqr.	4.90 × 10^−4^	2.27 × 10^−4^	2.16 × 10^−4^	1.23 × 10^−4^
*R*-Square (COD *)	0.992211058	0.814794067	0.578948913	0.570669812
Adj. *R*-Square	0.992204642	0.814631248	0.578541313	0.570250134
**Model**	**DB-c in ST**			
Plot	1st cycle	2nd cycle	3rd cycle	4th cycle
*A*	0	0	0	0
*B*	−0.1446	0.34435	0.22025	0.28696
*C*	4.26901	−0.52362	−0.26056	−0.43635
*D*	−2.58851	0.32061	0.15463	0.26718
Reduced Chi-Sqr.	8.54 × 10^−4^	3.00 × 10^−4^	1.29 × 10^−4^	2.08 × 10^−4^
*R*-Square (COD *)	0.996806629	0.83340502	0.887601356	0.83340502
Adj. *R*-Square	0.996804122	0.833274255	0.887513131	0.833274255

Note: *—Coefficient of determination; *x* is the shear angle in radian, *y* is the in-plane shear rigidity modules, and *A*, *B*, *C* and *D* are coefficients of the polynomial function.

Shear stress curves and the corresponding fitted curves of UD fabric, for example, are illustrated in Figure A1. The resultant functions were derived to obtain the in-plane shear rigidity modulus.
